# Tuberculosis

**Published:** 1894-11

**Authors:** 


### TUBERCULOSIS.

This disease seems to be an ever fruitful source of discussion at our meetings and in our journals. Have we acted as well as we have preached? In other words, have we not been led into generalization, and have not our conclusions quite often—too often—been that the United States Government and the different State Governments ought to employ veterinarians, at good salaries, to superintend the slaughter of animals evidently affected? We would suggest that it might be well to enter upon a line of investigation which would give us data, whereby it could be determined—at least approximately—what would have to be done, what the cost would be, and whether the result when obtained would justify such expense. A scientific investigation should have nothing to do with sentiment. The benefit of the whole country and the cost of such benefit should be the only things considered, provided the possibility of accomplishing such a matter as the control or eradication of tuberculosis can be demonstrated. The following points should be determined:

1st. By the National Government, the number, approximately, of cattle affected with tuberculosis.

2d. The value of such animals.

3d. The cost of inspection and disinfection.

4th. The number of deaths, annually, in people from tuberculosis.

5th. The social condition of the persons affected, that is, habit of living, whether in crowded tenements, sleeping with persons already affected, or if in the country, whether there is any relation between the infection in stable and household. When this information shall have been obtained, we can better discuss the question of control or eradication. We are most decidedly of the opinion, however, that measures for the eradication of tuberculosis will be fruitless, unless a strict quarantine be imposed upon persons as upon animals infected.

